# Trends in Severe Acute Respiratory Syndrome Coronavirus 2 (SARS‐CoV‐2) infection and vaccine antibody prevalence in a multi‐ethnic inner‐city antenatal population: A cross‐sectional surveillance study

**DOI:** 10.1111/1471-0528.17508

**Published:** 2023-04-27

**Authors:** Daria Andreeva, Carolyn Gill, Anna Brockbank, Joanna Hejmej, Fran Conti‐Ramsden, Katie J. Doores, Paul T. Seed, Lucilla Poston, the eLIXIR Partnership, David Edwards, Robert Stewart, Louise M Howard, Mark Ashworth, Jane Sandall, Francesca Happé, Andrew Shennan, Seeromanie Harding, Anne Greenough, Ingrid Wolfe, Lauren Carson, Amanda Grey, Cheryl Gillett, Claire Delaney‐Pope, Laura A Magee, Laura McFarlane, Melita Irving, Michael Absoud, Sarah Spring, Edward Barker, Amelia Jewell, Matthew Broadbent, Angela Flynn

**Affiliations:** ^1^ Department of Women and Children's Health, School of Life Course and Population Sciences King's College London London UK; ^2^ Department of Infectious Diseases, School of Immunology and Microbial Sciences King's College London London UK

**Keywords:** early pregnancy, infectious disease: virology, maternity services, medical disorders in pregnancy

## Abstract

**Objective:**

To determine severe acute respiratory syndrome coronavirus 2 (SARS‐CoV‐2) seroprevalence in pregnancy in an inner‐city setting and assess associations with demographic factors and vaccination timing.

**Design:**

Repeated cross‐sectional surveillance study.

**Setting:**

London maternity centre.

**Sample:**

A total of 906 pregnant women attending nuchal scans, July 2020–January 2022.

**Methods:**

Blood samples were tested for IgG antibodies against SARS‐CoV‐2 nucleocapsid (N) and spike (S) proteins. Self‐reported vaccination status and coronavirus disease 2019 (COVID‐19) infection were recorded. Multivariable regression models determined demographic factors associated with seroprevalence and antibody titres.

**Main outcome measures:**

Immunoglobulin G N‐ and S‐protein antibody titres.

**Results:**

Of the 960 women, 196 (20.4%) were SARS‐CoV‐2 seropositive from previous infection. Of these, 70 (35.7%) self‐reported previous infection. Among unvaccinated women, women of black ethnic backgrounds were most likely to be SARS‐CoV‐2 seropositive (versus white adjusted risk ratio [aRR] 1.88, 95% CI 1.35–2.61, *p* < 0.001). Women from black and mixed ethnic backgrounds were least likely to have a history of vaccination with seropositivity to S‐protein (versus white aRR 0.58, 95% CI 0.40–0.84, *p* = 0.004; aRR 0.56, 95% CI 0.34–0.92, *p* = 0.021, respectively). Double vaccinated, previously infected women had higher IgG S‐protein antibody titres than unvaccinated, previously infected women (mean difference 4.76 fold‐change, 95% CI 2.65–6.86, *p* < 0.001). Vaccination timing before versus during pregnancy did not affect IgG S‐antibody titres (mean difference −0.28 fold‐change, 95% CI −2.61 to 2.04, *p* = 0.785).

**Conclusions:**

This cross‐sectional study demonstrates high rates of asymptomatic SARS‐CoV‐2 infection with women of black ethnic backgrounds having higher infection risk and lower vaccine uptake. SARS‐CoV‐2 antibody titres were highest among double‐vaccinated, infected women.

## Abstract 2

This article includes Author Insights, a video abstract available at: https://emckclac‐my.sharepoint.com/:v:/g/personal/k1888594_kcl_ac_uk/ESBeVJ8VdMtMtDOaAflgfLIBoKJ6i7g8v‐OkeQEc8cyTnA?e=v3fXo8.

## Graphical abstract

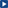

## 1 INTRODUCTION

Detrimental effects of severe acute respiratory syndrome coronavirus 2 (SARS‐CoV‐2) infection during pregnancy on maternal and neonatal outcomes have been widely reported. Pregnant women are at increased risk of severe complications including admission to intensive care, use of invasive ventilation and death compared with non‐pregnant women.^1^, ^2^ Maternal infection has been associated with adverse neonatal outcomes including preterm birth and neonatal admission.^3^ A recent observational study suggested that infection with SARS‐CoV‐2 Delta variant is associated with the highest risk of maternal adverse outcomes including oxygen support and mortality compared with the Omicron and pre‐Delta variants.^4^

Coronavirus disease 2019 (COVID‐19) vaccination programmes have significantly decreased complications from infection in the general population.^5^ Studies on vaccine efficacy in pregnant women were limited early in the pandemic because of the exclusion of this group from vaccine trials.^6^ However, subsequent studies have confirmed the benefit of vaccination in pregnancy. In the UK, vaccination of pregnant women has been strongly recommended by the Royal College of Obstetricians and Gynaecologists since 16 July 2021, although a review of all UK COVID‐19 critical care admissions of pregnant women between May and December 2021 demonstrated almost all admitted women (98%) were unvaccinated.^7^, ^8^ Recent studies suggest that booster doses during pregnancy can further increase antibody titres, providing better protection to the mother and fetus.^9^, ^10^, ^11^ Despite this, in 2022, a significant proportion of women remain unvaccinated at delivery in the UK (26.5% as of May 2022).^12^ The UK ‘Understanding Society’ COVID‐19 survey revealed that vaccine hesitancy in the general population was higher in women, those with lower education levels and individuals of black and Pakistani/Bangladeshi ethnic backgrounds.^13^ Although pregnant women were on the priority list for Autumn 2022 COVID‐19 booster doses, those from these ethnic minority backgrounds and of lower socio‐economic status may have been less likely to be vaccinated and therefore particularly vulnerable to the adverse effects of SARS‐CoV‐2 infection in pregnancy.^12^

Monitoring SARS‐CoV‐2 seroprevalence in pregnant women could contribute to the identification of individuals at risk of COVID‐19‐associated complications in antenatal populations. This study investigated early pregnancy SARS‐CoV‐2 seroprevalence in women enrolled for antenatal care in an inner‐city, multi‐ethnic population in London, UK and assessed associations with ethnicity, age, and socio‐economic and vaccination status. A secondary objective was to investigate the effect of vaccination status, vaccination timing (before or during pregnancy) and infection status on SARS‐CoV‐2 antibody titres using a repeated cross‐sectional study design.

## 2 METHODS

### 2.1 Sampling and recruitment

Women presenting for antenatal nuchal scans (11–15 weeks of gestation) at Guy's and St Thomas’ NHS Foundation Trust (GSTT) between July 2020 and January 2022 were invited to contribute to the Research Tissue Bank (COVID‐19 Substudy) of the Early Life Cross Linkage in Research (eLIXIR) programme.^14^ Invitation was by telephone and/or email before the appointment. All participants were provided with a patient information sheet. Following discussion with the research team, women who agreed to participate provided written informed consent.

### 2.2 Data collection

Participants' demographics, including maternal age, self‐reported ethnicity and postcodes, were obtained from antenatal electronic patient records (BadgerNet, CleverMed). Index of multiple deprivation was calculated from postcode Lower Super Output Area.^15^ Aggregate ethnic categories were defined according to the 2021 Census of England and Wales list (Office of National Statistics).^16^ Ethnic category groups are summarised in Table S1. COVID‐19 vaccination and infection status were self‐reported by women during their first antenatal appointment. Previous confirmed SARS‐CoV‐2 infection was defined as a self‐reported positive SARS‐CoV‐2 test result. Women recruited before COVID‐19 vaccination roll out (January 2021) were assumed to be unvaccinated. Women recruited from August 2021 onwards self‐reported their total number of COVID‐19 vaccinations.

Exact vaccination dates from the National Immunisation Management System (NIMS) were accessed for the subgroup of women who received SARS‐CoV‐2 vaccines at GSTT (*n* = 79). Cross‐validation of self‐reported vaccination status and NIMS data indicated absolute agreement in vaccination status in this subgroup (data not shown). No core outcome set was used.

### 2.3 Blood sample collection and processing

Following consent, participants donated a blood sample (6 mL clot‐activator tube), collected with routine venepuncture during their appointment. Samples were kept on ice after collection for a maximum of 5 hours, centrifuged (2500 *g*, 10 minutes, at 4°C), processed and split into aliquots on ice, then stored at −80°C until analysis.

### 2.4 ELISA

Samples were tested for IgG responses against SARS‐CoV‐2 nucleocapsid (N) and the spike (S) proteins using an in‐house enzyme‐linked immunosorbent assay (ELISA).^17^ High‐binding ELISA plates (Corning 3690; Corning, Ithaca, NY, USA) were coated with SARS‐CoV‐2 antigen (N or S) at 3 μg/mL (25 μL per well) in phosphate‐buffered saline (PBS), either overnight at 4°C or for 2 hours at 37°C. Wells were washed with PBS‐T (PBS with 0.05% Tween‐20) and blocked with 100 μL 5% milk in PBS‐T for 1 hour at room temperature. Wells were emptied and plasma diluted at 1:25 in 5% milk in PBS‐T was added and incubated for 2 hours at room temperature. Control reagents included CR3009 (2 μg/mL), CR3022 (0.2 μg/mL), negative control plasma (1:25 dilution), positive control plasma (1:25) and blank wells. Wells were washed five times with PBS‐T. Secondary antibody (goat‐anti‐human‐Fc‐alkaline phosphatase (AP) (1:1000) (109–055‐098; Jackson Laboratories, Bar Harbor, ME, USA) was added and incubated for 1 hour at room temperature. Wells were washed five times with PBS‐T and AP substrate (Sigma, St Louis, MO, USA) was added and read at 405 nm (AP).

### 2.5 Definition of infection and vaccination status

Clinical status was determined from antibody serology and self‐reported vaccination status (Table S2). Seropositivity (IgG) to SARS‐CoV‐2 S‐ and N‐proteins was defined as four‐fold increase or more in optical density (OD, 405 nm) above background.^17^ S‐protein antibodies are developed from both infection and vaccination. In contrast, N‐protein antibodies are developed only after infection, are highest following the acute phase and then subside. Irrespective of IgG N‐protein level change, women were classified as ‘negative’ if they were S‐protein seronegative.

In unvaccinated women, S‐protein seropositive individuals (N‐protein seropositive or seronegative) were classified as previously ‘infected and unvaccinated’. In vaccinated women, seropositivity to S‐protein was assumed to be a result of previous vaccination, in keeping with high seroconversion rates after vaccination.^18^ Therefore, S‐protein seropositive and N‐protein seronegative individuals reporting vaccination were classified as ‘history of vaccination’, whereas S‐ and N‐protein seropositive individuals reporting vaccination were classified as previously ‘infected and vaccinated’.

### 2.6 Statistical analysis

Binary regression models with log link were constructed to examine the association between participant demography and antibody serology, including presence of antibodies from infection or vaccination. Ethnicity, age and index of multiple deprivation were included in the multivariable model (Table 1). Risk ratios were reported with 95% confidence intervals. A one‐way analysis of variance (ANOVA) was used to test for the effect of vaccination, infection status and vaccination timing on IgG S‐protein antibody titres. For the vaccinated group, only women who had been double vaccinated were included in the ANOVA. A Bonferroni post‐hoc test determined which pair groups were significant. stata software, version 16.0 (StataCorp, College Station, TX, USA), was used for data analysis. The total number of patients in the study was determined by the number of participants for whom early pregnancy samples were available. Following Goodman and Berlin, we did not conduct a post‐hoc power calculation.^19^

**TABLE 1:** Maternal characteristics of the recruited participants, stratified by self‐reported vaccination status.

	Unvaccinated (*n* = 589)	Vaccinated (*n* = 371)
Age (years)	33 ± 5	34 ± 4
Gestational age at recruitment (weeks)	12.7 ± 2.0	12.5 ± 3.6
Infection status (self‐reported)		
Before pregnancy	49 (8%)	43 (12%)
During pregnancy	7 (1%)	12 (3%)
None	533 (91%)	316 (85%)
Ethnicity^a^		
Asian	53 (9%)	36 (10%)
Black	88 (15%)	25 (7%)
Mixed	38 (7%)	17 (4%)
White	372 (63%)	272 (73%)
Any other	38 (7%)	21 (6%)
Index of multiple deprivation^b^		
1 (most deprived)	117 (20%)	54 (15%)
2	257 (44%)	156 (42%)
3	120 (20%)	94 (25%)
4	61 (10%)	46 (12%)
5 (least deprived)	34 (6%)	21 (6%)
Vaccine doses		
1	–	94 (25%)
2	–	243 (66%)
3	–	20 (5%)
Unrecorded	–	14 (4%)

*Note*: Results shown are mean ± SD or *n* (%).

^a^ Ethnic category groupings are summarised in Table S1.

^b^ Scores were calculated for the region of residence, by fifths of the population. UK‐wide scores were developed from national English data relating to employment, income, education, health and housing domains.

### 2.7 Public and patient involvement

Public and patient involvement was incorporated throughout the development of the eLIXIR Partnership and is ensured in the decision‐making process of approving all eLIXIR projects through lay member representation on the eLIXIR Oversight Committee, which reviews and approves all projects using eLIXIR data.^14^

## 3 RESULTS

### 3.1 Study population description

Of 1552 women approached, 964 consented to participate in the study between 20 July 2020 and 21 January 2022. Blood samples were obtained from 960/964 (99.6%). Baseline participant demographics and self‐reported infection status stratified by self‐reported vaccination status are summarised in Table 1. In all, 371/960 (38.6%) women reported having been vaccinated with 263/960 (27.4%) having received at least two doses before enrolment. In the entire cohort, 92/960 (9.5%) women reported having a confirmed SARS‐CoV‐2 infection before pregnancy, and 19/960 (2.0%) reported a confirmed infection during pregnancy. The majority of women self‐identified as being of white ethnic backgrounds (67.1%), with those of black ethnic backgrounds being the second most common ethnic group (11.8%). The mean gestational age at recruitment was 12.6 weeks. The majority of the participants (60.8%) lived in the two most deprived Index of Multiple Deprivation quintiles. Self‐reported confirmed infections were significantly higher in the vaccinated group compared with the unvaccinated group (unadjusted risk difference 5.3%, 95% CI 1.0–9.6, *p* = 0.012). There were no missing data in participant demographics or self‐reported infection and vaccination status.

### 3.2 SARS‐CoV‐2 IgG S‐ and N‐antibody results and infection and vaccination status

In total, 471/960 (49.1%) women were classified as ‘negative’ for SARS‐CoV‐2 before infection and vaccination (S‐protein seronegative); 293/960 (30.5%) had an antibody profile consistent with ‘history of vaccination’ (S‐protein seropositive only with self‐reported vaccination). In all, 196/960 (20.4%) women were classified as previously infected (S‐protein seropositive in unvaccinated women or S‐ and N‐protein seropositive in vaccinated women). Of these, 29.6% (58/196) had self‐reported vaccination (‘infected and vaccinated group’), and 70.4% (138/196) did not (‘infected and unvaccinated group’). Only 70/196 (35.7%) women with serology consistent with past infection self‐reported previous confirmed infection before or during pregnancy. Twenty of the 371 (5.4%) women who self‐reported vaccination were S‐protein seronegative.

### 3.3 Changes in SARS‐CoV‐2 infection and vaccination status over time

**FIGURE 1:**
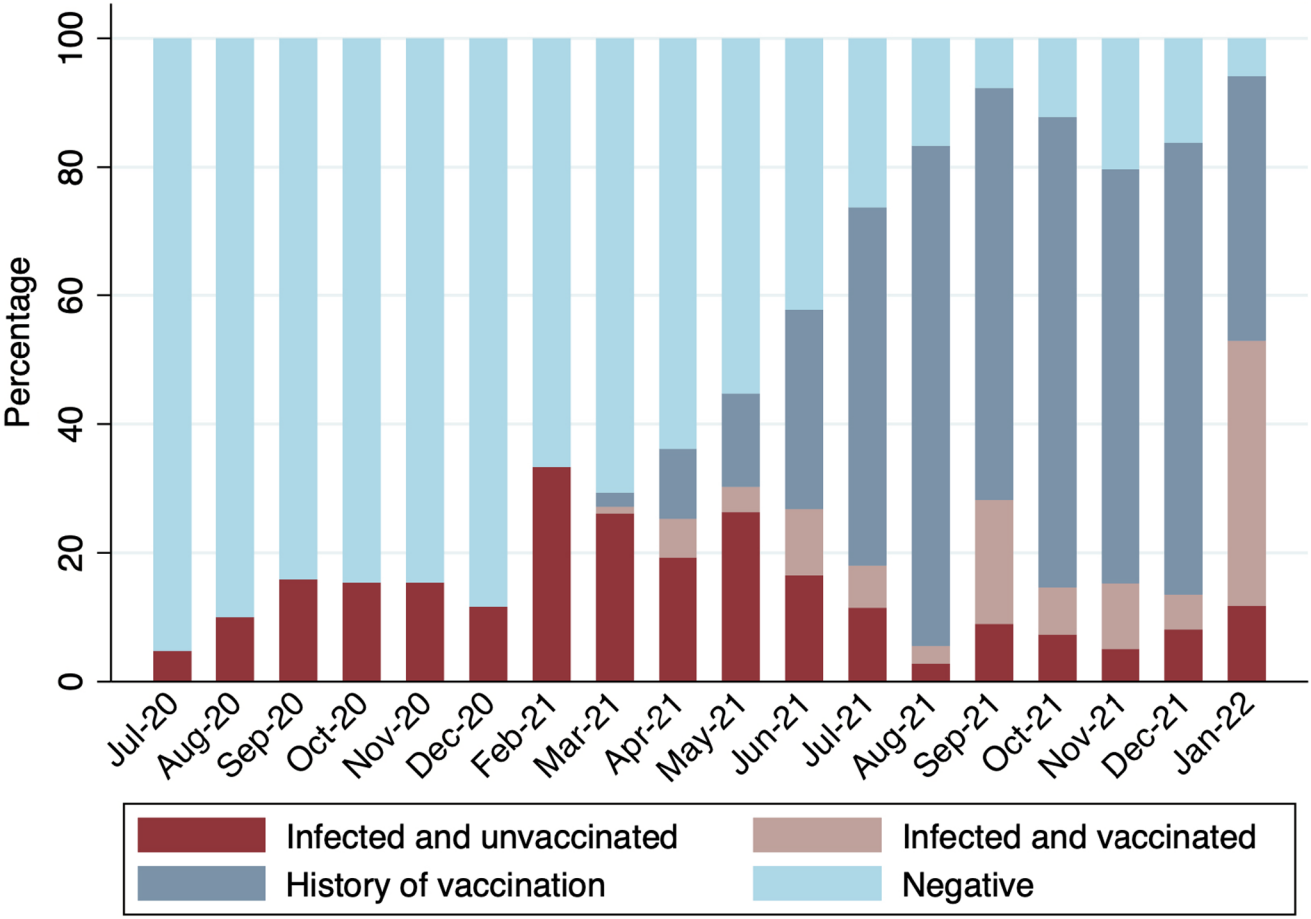
Previous infection and vaccination status (‘Infected and unvaccinated’, ‘Infected and vaccinated’, ‘History of vaccination’, and ‘Negative’) in the first trimester of pregnancy by month, from July 2020 until January 2022. Data exclude January 2021, as no participants were recruited during that month. Total number of participants, *n* = 960.

Previous infection and vaccination status in early pregnancy in the study cohort over time is shown in Figure 1. Monthly SARS‐CoV‐2 seroprevalence regardless of vaccination status (‘infected and vaccinated’ and ‘infected and unvaccinated’ groups) showed three peaks: February to June 2021 during the Alpha variant wave (average 27.5%), September 2021 during the Delta variant wave (28.2%) and January 2022 during the Omicron variant wave (52.9%). Since the start of the vaccination programme in December 2020, the monthly proportion of S‐protein seropositive vaccinated women regardless of infection status (‘history of vaccination’ and ‘infected and vaccinated’ groups) increased from 3/92 (3.3%) in March 2021, reaching a plateau of 58/72 (80.6%) in December 2021.

### 3.4 SARS‐CoV‐2 infection and vaccination status across maternal ethnic groups

**FIGURE 2:**
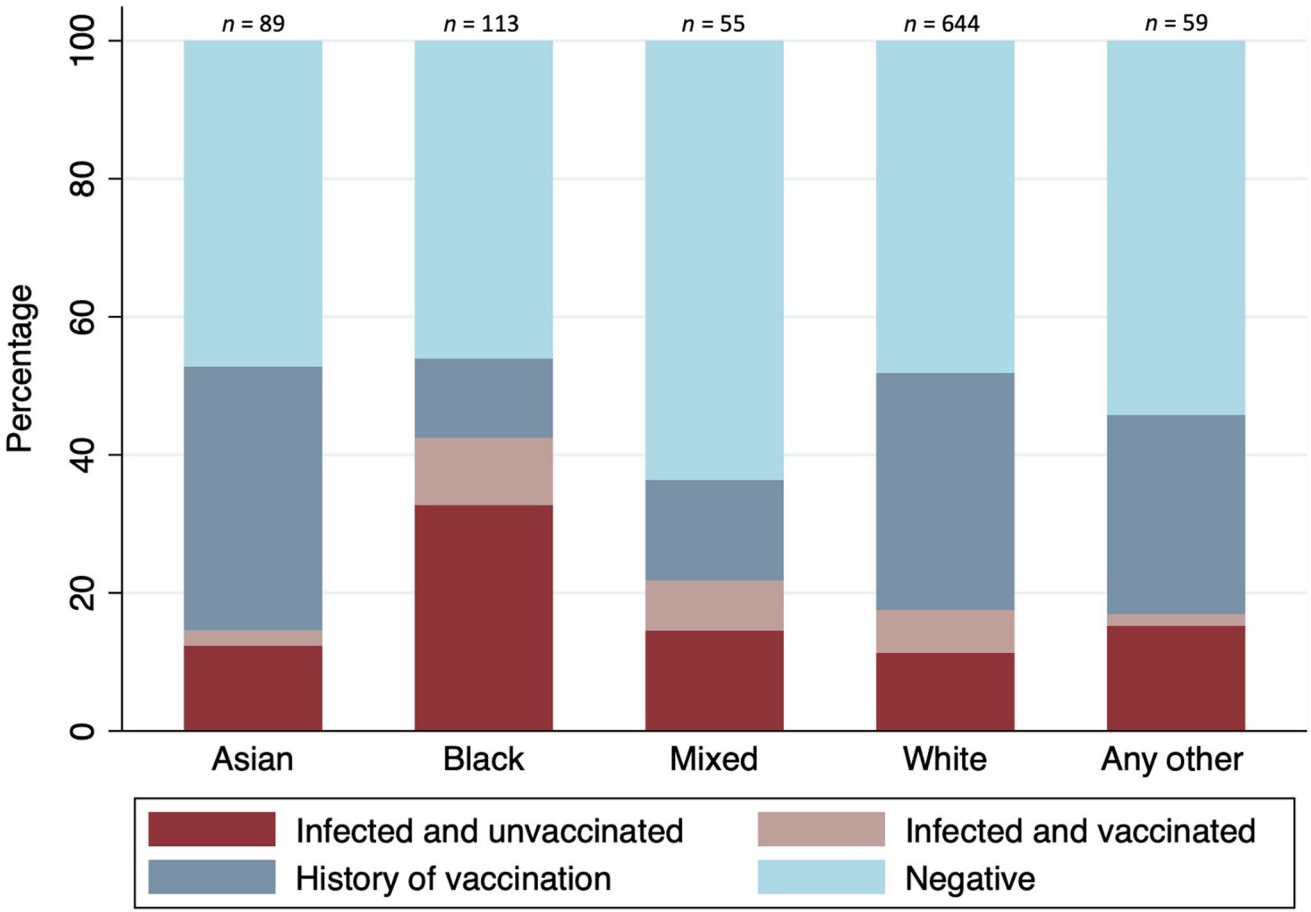
Previous infection and vaccination status (‘Infected and unvaccinated’, ‘Infected and vaccinated’, ‘History of vaccination’, and ‘Negative’) in the first trimester of pregnancy stratified by ethnicity. Total number of participants, *n* = 960.

Previous infection and vaccination status stratified by self‐reported ethnicity is shown in Figure 2. Regardless of vaccination status, women of black ethnic backgrounds had the highest SARS‐CoV‐2 seroprevalence (32.7% unvaccinated and 9.7% vaccinated) and the lowest overall profile consistent with ‘history of vaccination’ (11.5%). In contrast, Asian women had the highest proportion of profiles consistent with ‘history of vaccination’ (38.2%) and the lowest SARS‐CoV‐2 seroprevalence (12.4% unvaccinated and 2.2% vaccinated women).

In multivariable regression analysis of the unvaccinated cohort (*n* = 589) (Table 2), SARS‐CoV‐2 seroprevalence (seropositive to S ±N antigen) was significantly higher in women of black versus white ethnic backgrounds (adjusted risk ratio [aRR] 1.88, 95% CI 1.35–2.61, *p* < 0.001). There was no significant difference in SARS‐CoV‐2 seroprevalence when comparing women living in the most and least deprived areas. In multivariable regression analysis of the vaccinated cohort (*n* = 371), seropositivity to N‐antigen (indicating infection) was also higher in women of black ethnic backgrounds (aRR black versus white ethnic group 2.99, 95% CI 1.71–5.22, *p* < 0.001).

**TABLE 2:** Association between ethnicity, index of multiple deprivation scores and age with infection and vaccination status. Results are presented as risk ratios and 95% confidence intervals.

	Infection status^a^				Vaccination status^b^	
	Unvaccinated cohort (*n* = 589)		Vaccinated cohort (*n* = 371)		Whole cohort (*n* = 960)	
	RR (95% CI)	*p*	RR (95% CI)	*p*	RR (95% CI)	*p*
Ethnicity						
Asian	1.11 (0.64–1.94)	0.713	0.40 (0.10–1.59)	0.193	0.96 (0.73–1.26)	0.785
Black	1.88 (1.35–2.61)	<0.001	2.99 (1.71–5.22)	<0.001	0.58 (0.40–0.84)	0.004
Mixed	1.06 (0.56–2.02)	0.857	1.66 (0.68–4.08)	0.266	0.56 (0.34–0.92)	0.021
White	Reference	–	Reference	–	Reference	–
Any other	1.17 (0.64–2.12)	0.612	0.33 (0.05–2.25)	0.257	0.78 (0.53–1.15)	0.212
Deprivation^c^						
1 and 2	1.60 (0.94–2.73)	0.085	1.16 (0.58–2.30)	0.672	0.97 (0.78–1.22)	0.812
3	1.51 (0.83–2.75)	0.175	1.34 (0.63–2.84)	0.442	1.14 (0.89–1.45)	0.307
4 and 5	Reference	–	Reference	–	Reference	–
Age category (years)						
18–29	1.31 (0.97–1.79)	0.078	0.94 (0.46–1.92)	0.868	0.55 (0.41–0.74)	<0.001
30–39	Reference	–	Reference	–	Reference	–
40–49	0.78 (0.37–1.66)	0.520	1.18 (0.59–2.35)	0.645	1.15 (0.86–1.52)	0.347

Abbreviations: CI, confidence interval; RR, risk ratio.

^a^ Infection status is defined as seropositivity to IgG S ± N in unvaccinated women and seropositivity to IgG N in vaccinated women.

^b^ Vaccination status in the whole cohort was defined as self‐reported history of vaccination and seropositivity to IgG S.

^c^ Scores were calculated for the region of residence, by fifths of the population. UK‐wide scores were developed from national English data relating to employment, income, education, health and housing domains.

Women of black (aRR 0.58, 95% CI 0.40–0.84, *p* = 0.004) and mixed (aRR 0.56, 95% CI 0.34–0.92, *p* = 0.021) ethnic backgrounds in this cohort were less likely to have a history of vaccination with seropositivity to S‐antigen compared with women of white ethnic backgrounds. Younger women were also less likely to have a history of vaccination with seropositivity to S‐antigen (aRR 18–29 years age group to 30–39 years age group: 0.55, 95% CI 0.41–0.74, *p* < 0.001).

### 3.5 IgG S‐protein antibody titre differences between vaccinated and unvaccinated participants

A one‐way ANOVA test was performed to compare S‐protein IgG antibody titres between unvaccinated participants with evidence of recent previous infection (*n* = 78, ‘infected and unvaccinated’ group with positive IgG N‐antibodies), participants who had received at least two doses of the vaccine and were seropositive to S‐antigen (*n* = 217, ‘history of vaccination’ group with two or more vaccine doses) and those with two or more vaccine doses with evidence of infection (*n* = 38, ‘infected and vaccinated’ group with two or more vaccine doses, positive IgG N‐antibodies). Vaccinated women with missing vaccine dose data (*n* = 14 recruited before August 2021) were not included in this analysis. Given the nature of the study, timing of serum sampling in relation to vaccination and infection was not uniform.

Mean IgG S‐protein antibody fold‐change titres for unvaccinated infected women, those with previous history of two or more vaccination doses and those with two doses and evidence of infection were 10.29 (±5.08 standard deviation [SD]), 12.66 (±4.32 SD) and 15.05 (±4.37 SD), respectively. There was a statistically significant difference between the three groups (*F*_2, 330_ = 15.40, *p* < 0.001, see Fig. S1). A Bonferroni post‐hoc test confirmed that S‐protein antibody titres were significantly higher in participants who were infected and vaccinated with at least two doses, compared with the unvaccinated and infected group (mean difference [MD] 4.76, *p* < 0.001, 95% CI = 2.60–6.90). Participants who had a history of vaccination with at least two doses also showed significantly higher S‐protein antibodies compared with the unvaccinated and infected group (MD 2.37, *p* < 0.001, 95% CI 0.94–3.81), but significantly lower titres when compared with the vaccinated and infected group (MD −2.39, *p* = 0.009, 95% CI −4.29 to −0.47).

### 3.6 S‐level titre differences between women vaccinated before and during pregnancy

In participants vaccinated at GSTT for whom vaccination dates were accessed from NIMS (*n* = 79) a one‐way ANOVA was performed to compare S‐protein antibody fold‐change titres between participants vaccinated before (*n* = 19, mean S‐protein antibody titre 13.56 ± 4.22 SD) or during pregnancy (*n* = 60, mean S‐protein antibody titre 13.84 ± 4.94 SD). Although timing of serum sampling varied (median, interquartile range [IQR] days between vaccination and sampling: 128 [IQR 34] days for women vaccinated before pregnancy, 42 [IQR 45] days for women vaccinated during pregnancy), there was no statistically significant difference in S‐antibody titres between women vaccinated before or during pregnancy (MD −0.28, *F*_1, 77_ = 0.07, *p* = 0.785). In this sub‐group, women who had received two vaccination doses had sustained seropositivity against SARS‐CoV‐2 S‐antigen for up to 250 days after the last dose (Figure S2).

## 4 DISCUSSION

### 4.1 Main findings

Overall, we found SARS‐CoV‐2 seroprevalence from infection in this cross‐sectional study of pregnant women to be almost three times higher than self‐reported previous infection, suggesting high levels of asymptomatic infection in pregnancy. In contrast, there was high concordance between serology consistent with vaccination and self‐reported vaccination status, with only 5% of vaccinated women not testing positive for S‐antibodies after vaccination. The absence of antibodies in this small group could have been the result of the timing of vaccination in relation to sampling, with the majority of these women self‐reporting their last dose to be more than 6 months from sample collection, or because of very recent vaccination. Alternative explanations include an inadequate immune response to the vaccine, as has been observed in non‐pregnant cohorts.^18^, ^20^

Results showed significant ethnicity and age‐related disparities in SARS‐CoV‐2 infection and vaccination. Women of black and mixed ethnic backgrounds were less likely to be vaccinated (78% and 69% unvaccinated respectively). Regardless of vaccination status, ethnicity was strongly associated with SARS‐CoV‐2 seroprevalence from infection, highest among women of black ethnic backgrounds (42%). Double‐vaccinated and previously infected women developed the highest IgG S‐antibody titres. Vaccinated women, regardless of infection status, had significantly higher IgG S‐antibody titres compared with unvaccinated women who had been infected.

### 4.2 Strengths and limitations

To our knowledge, this is the first study to report SARS‐CoV‐2 seroprevalence in pregnant women living in London over the second and third waves of the pandemic, demonstrating a rise in infections during the third wave. The sample size (*n* = 960) provided adequate statistical power, as observed from the narrowness of the confidence intervals, and was comparable to a similar study conducted during the first wave in Oxford, UK (*n* = 1000).^21^ Participants recruited to this study were representative of the local population, with ethnic group proportions being similar to the antenatal population at GSTT.^14^

There are inherent limitations associated with the use of antibody serology. As a result of the temporaneous waning of SARS‐CoV‐2 N‐protein IgG antibodies following viral infection, it was not possible to determine whether vaccinated individuals with positive S‐protein antibody titres only had previously been infected. Timing of serum sampling in relation to vaccination and infection varied between each woman, complicating comparison of antibody titres. Moreover, lack of seroconversion following infection in some participants may have led to underestimation of seroprevalence. As vaccination dates were only available in a subgroup of women, analysis of antibody titres between women vaccinated before and after pregnancy was conducted on a smaller sample.

### 4.3 Interpretation

Ascertainment of COVID‐19 serology over an 18‐month time period in this study allows for comparison with UK government SARS‐CoV‐2 population infection data. As IgG antibodies require 2–3 weeks to develop, the first peak in antibodies from infection, observed between February and June 2021 in this study, coincides with NHS Test and Trace data showing a peak in the number of UK infections during the second wave following the emergence of the Alpha variant in January 2021.^22^, ^23^ The UK Office for National Statistics reported a third spike in infections in July 2021, attributed to the new Delta variant. The peak in antibodies from infection observed in our data in September 2021 coincides with the start of this third spike, whereas the higher proportion of pregnant women booking for antenatal care in January 2022 with antibodies from infection observed in our study is likely to be associated with the Omicron variant that appeared in December 2021.^24^

The overall SARS‐CoV‐2 seroprevalence of 20.4% in early pregnancy observed is markedly higher than the 5.3% reported in a pregnant study cohort in Oxford, UK during the first wave of the pandemic.^21^ Lower seroprevalence rates were also observed during the second wave in a Scottish national study reporting an estimate of 11.3% among the pregnant cohort.^25^ In this study, the increase in seroprevalence following the first and second waves coincides with the relaxation in governmental preventive measures against viral spread. Studies conducted internationally in pregnant women living in large cities report similar seroprevalence to the figure reported here (21.4% in Madrid, Spain and 16.1% in New York City, USA).^26^, ^27^ Despite this, high rates of asymptomatic infection were a common finding among several studies, including our own, highlighting the importance of monitoring seroprevalence.^26^, ^27^, ^28^, ^29^

The association between demographics and vaccination uptake reported here can be compared to national data from pregnant women collected between August and October 2021.^11^ During this 3‐month period, lower vaccination rates at delivery were reported in women of black ethnic background (85.7% unvaccinated), those living in the most deprived areas (81.7% unvaccinated) and the younger age cohort up to 29 years old (80.8% unvaccinated). Our data from July 2020 to January 2022 showed similar disparities, with lower vaccination uptake and higher rates of COVID‐19 infection in women of black ethnic backgrounds and in younger women. Differences in infection across ethnic backgrounds are likely to be multifactorial, with occupation, lack of personal protection equipment, comorbidities and socio‐economic circumstances being strong contributors.^30^, ^31^

Our data indicate the need for targeted action to improve vaccine acceptance and uptake among pregnant women from ethnic minority backgrounds and in younger age groups. Safety concerns around vaccinations during pregnancy and reduced access to vaccines significantly contribute to hesitancy in minority ethnic groups.^32^, ^33^, ^34^ Lack of knowledge among healthcare professionals on vaccine safety and efficacy is an additional limiting factor to uptake in pregnant women.^32^, ^33^ With the ongoing booster rollout programme, interventions, including healthcare staff education on vaccine safety in pregnancy and easier access through antenatal care, are needed to address these disparities.

We found that pregnant women who were double vaccinated and previously infected developed the highest IgG S‐protein antibodies titres. Vaccinated women, regardless of infection status, had significantly higher antibody titres compared with unvaccinated women who were infected. These findings are supported by previous studies which report vaccine‐induced immune responses being significantly greater compared with natural infection.^9^, ^35^ In this study, the titres of antibodies following vaccination were similar between women vaccinated before or during pregnancy, consistent with previous research.^35^, ^36^ As double‐vaccinated women with accurate dates of vaccination showed sustained seropositivity to S‐antibody for up to 250 days after the second dose, this implies a lasting degree of immunity.

We report high rates of asymptomatic SARS‐CoV‐2 infection in UK inner city pregnant women and high rates of infection among women of black ethnic background. The lower vaccine uptake in this group, in younger women and in women of mixed ethnic background, indicates increased vulnerability to severe infection and a need for targeted action to enhance vaccine uptake in booster programmes. Benefits of vaccination for pregnant women are indicated by the highest IgG antibody titres against S‐protein in double‐vaccinated women with a previous infection, and higher titres among vaccinated women without a previous infection compared with unvaccinated infected women. Timing of vaccination, whether before pregnancy or in the first trimester, did not affect antibody titres when measured at 10–12 weeks of pregnancy.

## AUTHOR CONTRIBUTIONS

The authors' responsibilities were as follows: LP and CG designed the research; DA, CG, AB, JH, KJD, PTS and FC‐R analysed the samples and data; DA wrote the first draft of the manuscript which was reviewed by KJD, LP, FC‐R and DA. LP had primary responsibility for the final content of the manuscript. All authors read and approved the final manuscript.

## FUNDING INFORMATION

This work was supported by eLIXIR Partnership developed by an MRC Partnership Grant (MR/P003060/1). The eLIXIR platform is also part‐supported by the National Institute for Health Research (NIHR) Biomedical Research Centre at the South London and Maudsley NHS Foundation Trust and King's College London. The eLIXIR research tissue bank was supported in part by the National Institute for Health Research (NIHR) Biomedical Research Centre at Guy's and St Thomas' NHS Foundation Trust and King's College London. Paul T Seed and Lucilla Poston are partly funded by Tommy's (Registered charity no. 1060508) and by ARC South London (NIHR).

## CONFLICT OF INTEREST STATEMENT

None declared.

## ETHICS APPROVAL

The eLIXIR Partnership received ethical approval for the tissue research tissue bank from the Cambridge East REC (18/EE/0120) submitted on 26 June 2020.

## Supporting information


Appendix S1

Data S1

Data S2

Data S3

Data S4

Data S5

Data S6

Data S7

Data S8

## Data Availability

The data accessed by eLIXIR remain within an NHS firewall, and governance is provided by the eLIXIR Oversight Committee reporting to relevant information governance clinical leads. Subject to these conditions, data access and sample access are encouraged and those interested should contact the eLIXIR Principal Investigator (Professor Lucilla Poston; lucilla.poston@kcl.ac.uk).

## APPENDIX 1

### Members of the eLIXIR Partnership

Professor Lucilla Poston, Tommy's Professor of Maternal & Fetal Health, Department of Women and Children's Health, School of Life Course and Population Sciences, King's College London. Professor David Edwards, Chair in Paediatrics & Neonatal Medicine, Department of Perinatal Imaging and Health, King's College London. Neonatal Consultant at Guy's and St. Thomas' NHS Foundation Trust. Professor Robert Stewart, Professor of Psychiatric Epidemiology & Clinical Informatics, Department of Psychological Medicine, Institute of Psychiatry, Psychology and Neuroscience, King's College London and NIHR Maudsley Biomedical Research Centre, South London and Maudsley NHS Foundation Trust, London. Consultant Psychiatrist at South London and Maudsley NHS Foundation Trust, London. Professor Louise M Howard, Professor of Women's Mental Health, Section of Women's Mental Health, Institute of Psychiatry, Psychology and Neuroscience, King's College London and NIHR Maudsley Biomedical Research Centre, South London and Maudsley NHS Foundation Trust, London. Consultant Psychiatrist at South London and Maudsley NHS Foundation Trust, London. Dr Mark Ashworth, Reader of Primary Care, Department of Population Health Sciences, School of Life Course and Population Sciences, King's College London. Professor Jane Sandall, Professor of Social Science & Women's Health, Department of Women and Children's Health, School of Life Course and Population Sciences, King's College London. Professor Francesca Happé, Professor of Cognitive Neuroscience, Social, Genetic and Developmental Psychiatry Centre, Institute of Psychiatry, Psychology and Neuroscience, King's College London. Professor Andrew Shennan, Professor of Obstetrics, Department of Women and Children's Health, School of Life Course and Population Sciences, King's College London and Obstetric Consultant at Guy's and St. Thomas' NHS Foundation Trust. Professor Seeromanie Harding, Professor of Social Epidemiology & Nutrition, Department of Diabetes, School of Life Course and Population Sciences, King's College London. Professor Anne Greenough, Professor of Neonatology and Clinical Respiratory Physiology, Department of Women and Children's Health, School of Life Course and Population Sciences, King's College London and Neonatal Consultant at King's College Hospital NHS Foundation Trust. Dr Ingrid Wolfe, Clinical Senior Lecturer, Department of Women and Children's Health, School of Life Course and Population Sciences, King's College London and Consultant in Children's Public Health Medicine and Director of the Evelina London Children's Healthcare. Dr Lauren Carson, Visiting Research Associate, Department of Psychological Medicine, Institute of Psychiatry, Psychology and Neuroscience, King's College London. Ms Amanda Grey, Lay member of the eLIXIR Oversight Committee. Dr Cheryl Gillett, Head of Tissue Banking, Department of Comprehensive Cancer Centre, School of Cancer & Pharmaceutical Sciences, King's College London. Ms Claire Delaney‐Pope, Head of Information Governance, South London and Maudsley NHS Foundation Trust, London. Professor Laura A Magee, Professor of Women's Health, Department of Women and Children's Health, School of Life Course and Population Sciences, King's College London. Ms Laura McFarlane, Director of the LEAP Programme, National Children's Bureau. Dr Melita Irving, Clinical Genetic Consultant at Guy's and St. Thomas' NHS Foundation Trust. Dr Michael Absoud, Paediatric Consultant at Evelina London Children's Healthcare. Mr Paul Seed, Reader in Medical Statistics, Department of Women and Children's Health, School of Life Course and Population Sciences, King's College London. Ms Sarah Spring, Lay member of the eLIXIR Oversight Committee. Dr Edward Barker, Reader, Centre for Population Neuroscience and Precision Medicine, Institute of Psychiatry, Psychology and Neuroscience, King's College London. Ms Amelia Jewell, Clinical Data Linkage Service Lead, NIHR Maudsley Biomedical Research Centre, South London and Maudsley NHS Foundation Trust. Mr Matthew Broadbent, CRIS Clinical Informatics Lead, NIHR Maudsley Biomedical Research Centre, South London and Maudsley NHS Foundation Trust, London. Dr Angela Flynn, Lecturer in Nutritional Sciences, Faculty of Life Sciences and Medicine, School of Life Course and Population Sciences, King's College London.
