# Supplementary material for: Trends in Severe Acute Respiratory Syndrome Coronavirus 2 (SARS‐CoV‐2) infection and vaccine antibody prevalence in a multi‐ethnic inner‐city antenatal population: A cross‐sectional surveillance study
Source: BJOG. 2023 Apr 27;130(9):1135–44. doi: 10.1111/1471-0528.17508 (PMC10718194; doi:10.1111/1471-0528.17508)
Supplement: Supplementary file 1 — Appendix S1 [file BJO-130-1135-s008.docx]

**Table S1.** List of ethnic groups and subgroups used in the study, as per 2021 Census of England and Wales guidelines.^13^

| Groups | Subgroups |
| --- | --- |
| Asian | - Indian - Pakistani - Bangladeshi - Chinese - Any other Asian background |
| Black | - Caribbean - African - Any other Black, Black British, or Caribbean background |
| Mixed | - White and Black Caribbean - White and Black African - White and Asian - Any other Mixed or multiple ethnic background |
| White | - English, Welsh, Scottish, Northern Irish or British - Irish - Gypsy or Irish Traveller - Roma - Any other White background |
| Any Other | - Arab - Any other ethnic group |

**Table S2.** Definition of clinical status categories determined by antibody serology and self-reported vaccination status.

|  | **IgG S serology** | **IgG N serology** | **Clinical status** |
| --- | --- | --- | --- |
| **Unvaccinated** | - | -/+ | Negative^1^ |
|  | + | - | Infected and unvaccinated^2^ |
|  | + | + | Infected and unvaccinated^3^ |
| **Vaccinated** | - | -/+ | Negative^1^ |
|  | + | - | History of vaccination |
|  | + | + | Infected and vaccinated |

+: ≥ 4 fold change from background (seropositive)

-: < 4 fold change from background (seronegative)

-/+: any fold change from background

^1^Positive IgG N alone was considered as spurious result/error

^2^Unvaccinated women with IgG S positive and IgG N negative status were considered positive for previous infection more than 4 months prior to sample collection

^3^Unvaccinated women with IgG S and N positive status were considered positive for previous recent infection within 4 months of sample collection


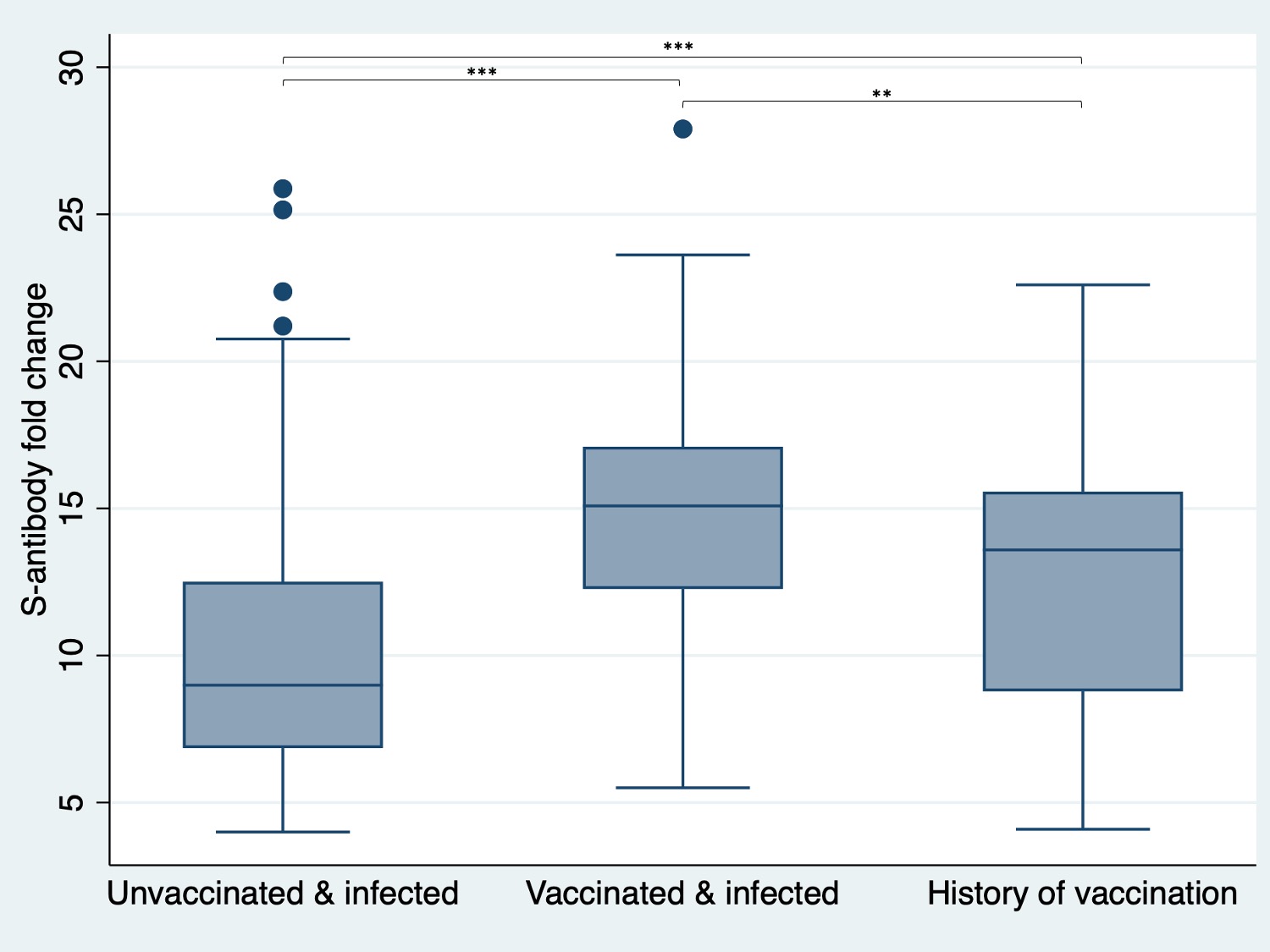


**Figure S1.** Boxplot of IgG S-protein antibody titers comparing unvaccinated women with IgG S and N positive antibodies indicating recent infection (“Unvaccinated & infected”, n = 78), women with a history of vaccination with at least two vaccine doses and evidence of infection (“Vaccinated & infected”, n = 38), and history of vaccination with at least two doses (“History of vaccination”, n = 217). **: P<0.01, ***: P<0.001.


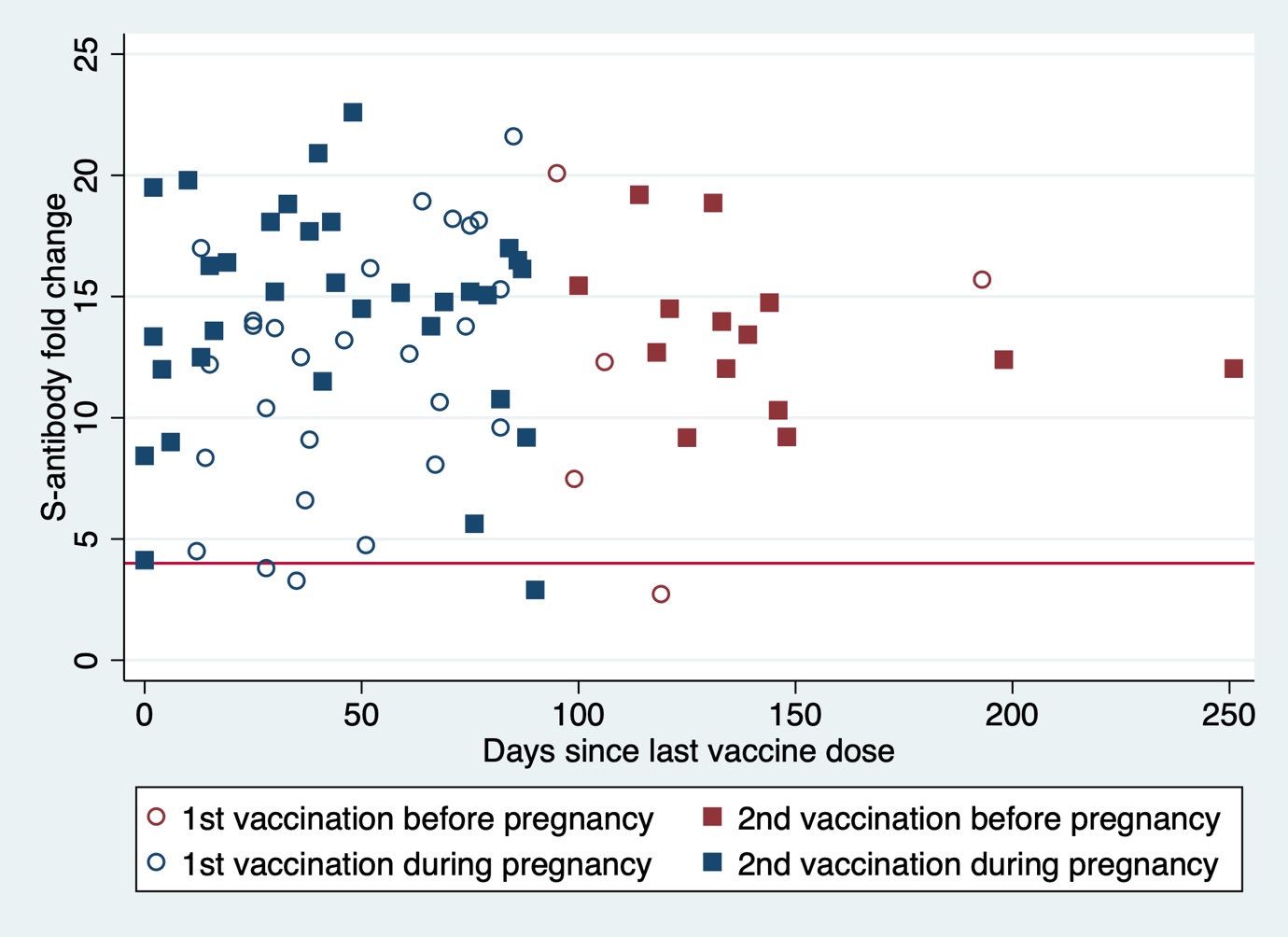


**Figure S2.** IgG S-antibody fold change titers plotted against time between last vaccination dose and serum sample collection. *Participants were split into groups based on timing and total number of vaccination doses received: women with only 1 dose received before pregnancy (“1^st^ vaccination before pregnancy”, n = 5) or during pregnancy (“1^st^ vaccination during pregnancy”, n = 27) and women with a total of 2 doses with latest received before pregnancy (“2^nd^ vaccination before pregnancy”, n = 14) or during pregnancy (“2^nd^ vaccination during pregnancy”, n = 33). The red line represents threshold used in the study to determine seropositivity (S-antibody fold change above 4).*
